# Cementitious Composites Reinforced with Magnetically Oriented Steel Microfibers: Mechanical Properties, Deformability and Fracture Propagation

**DOI:** 10.3390/ma18204739

**Published:** 2025-10-16

**Authors:** Maciej Kaźmierowski, Marta Kadela, Michał Kordasz, Filip Chyliński, Roman Jaskulski, Michał Drzazga, Małgorzata Wydra, Kacper Marchwicki, Andrzej Cińcio

**Affiliations:** 1 Department of Civil Engineering, Faculty of Environmental Engineering and Geodesy, Wrocław University of Environmental and Life Sciences, ul. Norwida 25, 50-365 Wrocław, Poland; roman.jaskulski@upwr.edu.pl (R.J.); michal.drzazga@upwr.edu.pl (M.D.); kacper.marchwicki@upwr.edu.pl (K.M.); 2 Building Research Institute (ITB), ul. Filtrowa 1, 00-611 Warsaw, Poland; f.chylinski@itb.pl; 3 Department of Graphics, Computer Vision and Digital Systems, Faculty of Automatic Control, Electronics and Computer Science, Silesian University of Technology, ul. Akademicka 2A, 44-100 Gliwice, Poland; mkordasz@polsl.pl; 4 Faculty of Civil Engineering, Mechanics and Petrochemistry, Warsaw University of Technology, ul. Łukasiewicza 17, 09-400 Płock, Poland; malgorzata.wydra@pw.edu.pl; 5 Department of Civil and Environmental Engineering, University of Michigan, 2350 Hayward St, Ann Arbor, MI 48109, USA; 6 Department of the Theory of Building Structures, Faculty of Civil Engineering, Silesian University of Technology, ul. Akademicka 2A, 44-100 Gliwice, Poland; andrzej.cincio@polsl.pl

**Keywords:** cement mortar, steel fibers, magnetic field, electromagnet, flexural strength, compressive strength, deformability, digital image correlation, fracture

## Abstract

The aim of the manuscript is to analyze the influence of the magnetic orientation of steel microfibers (length 13 mm, diameter 0.2 mm) on the mechanical properties and fracture propagation of cementitious composites. The series varied in terms of the volumetric content of the fibers, 0%, 1% and 2% (*V_f_*), and the orientation variant, random (S) or magnetic (S-M, *B* = 80 mT). Three-point bending tests were performed with force-deflection curve (*F-δ*) registration. The flexural tensile strength (*f_ct,_ _fl_*), the flexural elastic modulus (*E_f_*), the work of fracture up to a specified residual load level *(W_f_*) and deflection level (W_f_*), as well as the compressive strength (*f_c_*) were determined. The improvement of the mechanical properties was noted for magnetically oriented fibers in reference to random arrangement (*f_ct,_ _fl_*: 90–133%; *f_c_*: 12–34%; W_f_*: 98–146%). The efficiency factor (*η_X_*) was introduced to determine the change in property per fiber content unit, which enabled comparison regardless of the fiber dosage. As the higher *η_X_* values were determined for 1% content (e.g., *f_ct,_ _fl_* equal to 133%/p.p for *V_f_* = 1% and 45%/p.p for *V_f_* = 2%), further increase in dosage was expected to cause reduced improvement. Different fracture mechanisms were noted for S and S-M composites by means of the Digital Image Correlation method.

## 1. Introduction

The progressive urbanization and globalization are promoting the design of high-rise buildings with complexed geometry of structural components. The development of new technologies is needed, e.g., prefabrication or 3D printing [1], which fulfill the assumptions of the sustainable building movement [2]. Consequently, the necessity of new materials development arises, focusing on the design and technological solutions increasing mechanical properties of cementitious composites. One of the solutions is the use of dispersed reinforcement. Examples can be found in the literature considering the use of various fiber types: steel [3,4,5], polypropylene [6], glass [5,7,8], carbon [9,10], basalt [11,12] or organic such as cellulose [13].

However, steel fibers addition is still one of the most widespread improvement methods that aim to increase the flexural strength and fracture resistance of the cementitious composite [14,15,16]. The extent of this improvement depends on the properties of singular fibers. The following properties are important to be considered: slenderness (*λ*) and shape of the fibers, volumetric dosage (*V_f_*) [17], bond to the cementitious matrix (resulting from adhesion, friction and mechanical anchoring [18]) and the three-dimensional arrangement of the fibers in composite (location and orientation) [19].

The typical value of the fiber orientation coefficient for the composite with randomly oriented fibers is about 0.42, while for the fiber-reinforced composites prepared with the use of magnetic field, even values over 0.9 can be determined [20,21,22]. The concept of magnetic field use to orient steel fibers was first introduced in the patents by Miller and Björklund [23] and Svedberg [24]. Multiple research investigations demonstrated that the use of magnetic field with the induction values from several dozen to several hundred mT can improve the mechanical properties of steel fiber reinforced cementitious composite (SFRCCs), see Table 1. It can be observed that the increase in compressive and tensile strength values is about 10–30%, and for the properties related to cracking resistance (e.g., fracture energy), it is 10–336%. It has also already been proven that magnetic orientation of fibers enables the reduction in fiber amount by about 40%, still retaining mechanical properties comparable to composites with randomly oriented fibers [25]. Also, different cracking mechanism can be noted for SFRCCs with magnetically oriented fibers in reference to those with randomly oriented fibers, ranging from local cracking and brittle damage to more spread and multipath forms of degradation as presented in [25,26,27].

The efficiency of the fiber orientation depends on mix viscosity [29], compaction methods, fiber slenderness and the time of the magnetic field exposure [41,42,43]. Wijffels et al. [29] used the magnetic field with the 20 mT induction for the time duration of 30 s, while in the research conducted by Javahershenas et al. [34], it was 500 mT and from 60 s. to 180 s. However, Ferrandez et al. [32] stated that too high induction may lead to aggregate segregation and excessive fibers gathering, resulting in heterogeneity of the structure. Additionally, Mu et al. [35] have proven that, in such a case, more uneven distribution of fibers can be noted in the cross sections of bent composites.

The considerations described above led to the conclusion that there is a necessity of carrying out further investigations on cementitious composites reinforced with magnetically oriented steel fibers (length < 30 mm). Such investigations constitute only about 30% of all summarized in Table 1, while only two of them pertain to cementitious mortars.

Da Silva Brito et al. [26] investigated the influence of microfibers’ orientation induced by magnetic field in high-performance cement mortar (*f_c_* = 81–117 MPa). Three-point bending tests carried out on the specimens with the dimensions of 40 mm × 40 mm × 160 mm confirmed that the magnetic orientation of the fibers resulted in *f_ct, fl_* increase by about 72% (*V_f_* = 1%), 23% (*V_f_* = 2%) and 73% (*V_f_* = 5%) in reference to the specimens with random orientation. Abrishambaf et al. [30], on the other hand, analyzed the influence of magnetic orientation of micro-reinforcement in ultra-high performance cementitious mortar, but only under uniaxial tensile strength tests. As a result, the experimental gap can be seen in the lack of a complex analysis of such composites.

Therefore, the aim of this article is the assessment of the influence of microfibers with the length of 13 mm and diameter of 0.2 mm (*λ* = 65) oriented with the use of magnetic field on the behavior of the reinforced cementitious composite. Two states of fiber orientations were analyzed: random (S) and magnetically oriented (S-M). Investigations were carried out for two volumetric dosages of fibers (*V_f_*): 1% and 2%. The results were also compared with the reference specimens without fibers.

The broad recognition of the performance characteristics describing the composites submitted to bending was enabled owing to the force deflection (*F-δ*) curve registration during the tests. As a result, the following parameters were determined: flexural tensile strength (*f_ct, fl_*), flexural elastic modulus (*E_f_*) and the work of the fracture in two variants: (i) with an assumed residual force level (*W_f_*) and (ii) with an assumed end deflection value (W_f_*). Compressive strength tests were performed as well. Efficiency factor *η_X_* was introduced to assess the influence of magnetic orientation on the analyzed properties, regardless of fiber dosage. The propagation and location of the fractures were described based on the principal tensile strain (*ε*_1_) maps obtained from the use of the Digital Image Correlation method.

## 2. Materials and Methods

### 2.1. Materials and Specimen Preparation

#### 2.1.1. Components

Mortar specimens were prepared out of cement, sand, water and admixture. CEM I 52.5 R class cement as per the [44] standard was used, with a specific surface area of 4210 cm^2^/g (value determined on Blaine’s manual apparatus [45]) and a specific density of *ρ* = 3050 kg/m^3^. The sand, fraction of 0–2 mm, as per the [46] standard, and a commercial superplasticizing admixture (SP) Chryso^®^ (Saint-Gobain Construction Chemicals, Courbevoie, France), based on polycarboxylates, with the total amount of solid content 30% and *ρ* = 1 120 kg/m^3^, were used. Water to cement ratio was equal to 0.5. In Table 2, the mix compositions for each series are presented.

**Figure 1:**
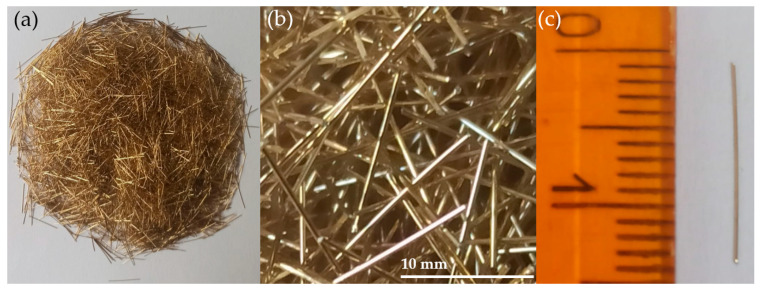
Steel fibers used in this research: (**a**) batch of fibers; (**b**) magnified view of fibers; (**c**) singular fiber (own photos).

Straight, smooth fibers from cold-drawn wire (high carbon steel) with a density of 7850 kg/m^3^, Young’s modulus of 200 GPa and tensile strength of 2000 Mpa (Figure 1) were used. The steel fibers (SF) had a length of 13 mm and a diameter of 0.2 mm (*λ* = 65).

#### 2.1.2. Mix Preparation

**Figure 2:**
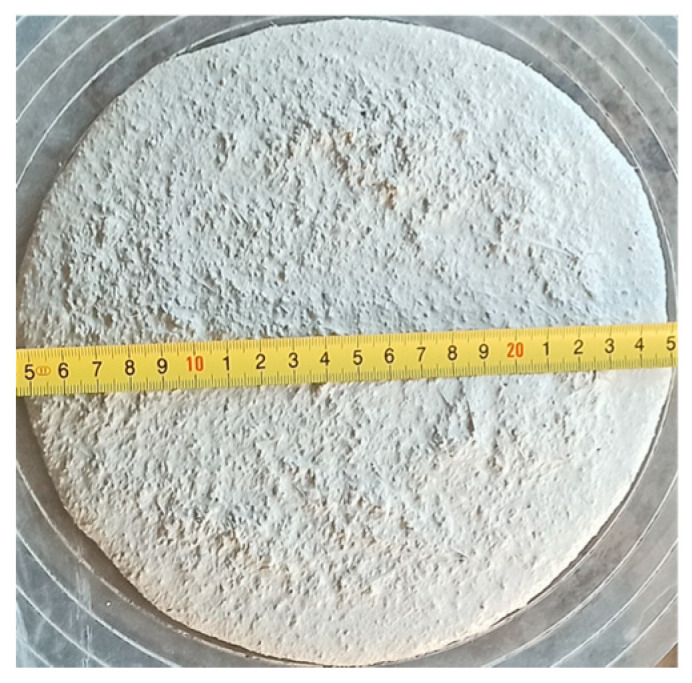
Consistency measurement of fresh cement mortar using the flow table method (own photo).

The mixing process was performed as per the procedure described in the [46] standard, with the modifications described below. Cement and standard sand were homogenized for 60 s in the laboratory mixer (UTEST Material Testing Equipment, Ankara, Turkey). Then, the water mixed with superplasticizer was dosed for 120 s. The steel fibers were added and mixed for 60 s to ensure their distribution in the composite mix. The ingredients were measured with the accuracy of ±1 g (cement, sand, water) and ±0.01 g (fibers, superplasticizer). To limit the influence of varying content of fibers on the rheological properties of cementitious composites, the same consistency was assumed for all the mixes. The different superplasticizer dosages (Table 2) enabled the achievement of this goal. The consistency of fresh mortar was controlled on the flow table (PREMA, Kielce, Poland) according to the [47] standard. For all series, repeatable results of the mix spread were determined equal to 20 ± 0.5 cm (Figure 2).

The ready mixes were poured into rectangular molds with dimensions of 40 mm × 40 mm × 160 mm. Three specimens were prepared for each series. After forming, the specimens of Ref, S-1 and S-2 series were compacted using mechanical vibrations, providing proper mortar compaction as well as favorable dispersion and orientation of the fibers. The specimens of S1-M and S2-M series were placed inside the electromagnetic coil, in which magnetic field was generated for 30 s. Simultaneously, those specimens were also submitted to mechanical vibrations. The specimens were demolded after 24 h and cured according to the [46] standard.

#### 2.1.3. Electromagnet and Magnetic Field: Setup, Modeling and Verification

The test stand design aimed to set up the electromagnet able to generate magnetic field with regulated induction in the range from 0 to 150 mT.

**Figure 3:**
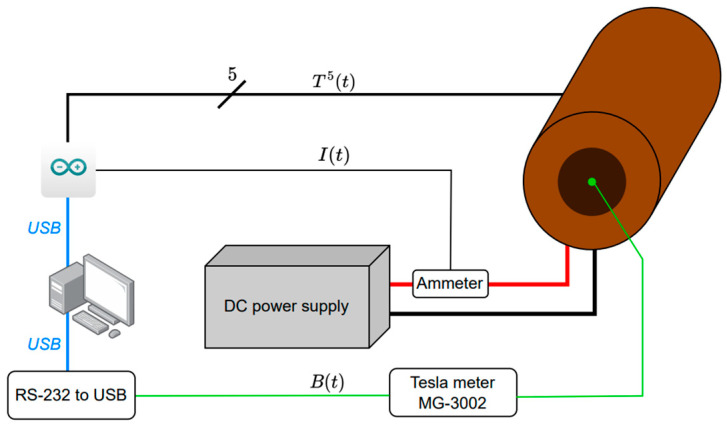
Block diagram of the power supply system and measurement systems for magnetic field induction *B*(*t*), electric current intensity *I*(*t*) and temperature *T*^7^(*t*).

Solutions were introduced to enable temperature measurements of the winding in seven spots (T^7^), ensuring that the research was carried out in a thermally safe environment and under repeatable conditions. The coil was powered from a programmable source of direct current, with fluent regulation of voltage and electric current intensity. The MG-3002 (Lutron, Taipei, Taiwan) sensor with RS-232 interface was used to measure the magnetic field induction. The wiring diagram of the test stand is shown in Figure 3.

Theoretical and numerical analyses were performed to ensure the repeatability and control of the magnetic field influence. The starting point was the general form of the Biot–Savart law, describing the magnetic field induction in the point P caused by the conductor element, in which the current I flows—Equation (1).

(1) $$d\vec{B}=\frac{\mu_{0}}{4\pi }\frac{Id\vec{l}\times \hat{r}}{r^{3}}$$

where

μ_0_—the magnetic permeability of the vacuum; *I*—electric current intensity; $d\vec{l}$—tangent vector to the conductor element at a given point; *r*—distance between the point P and conductor element.

**Figure 4:**
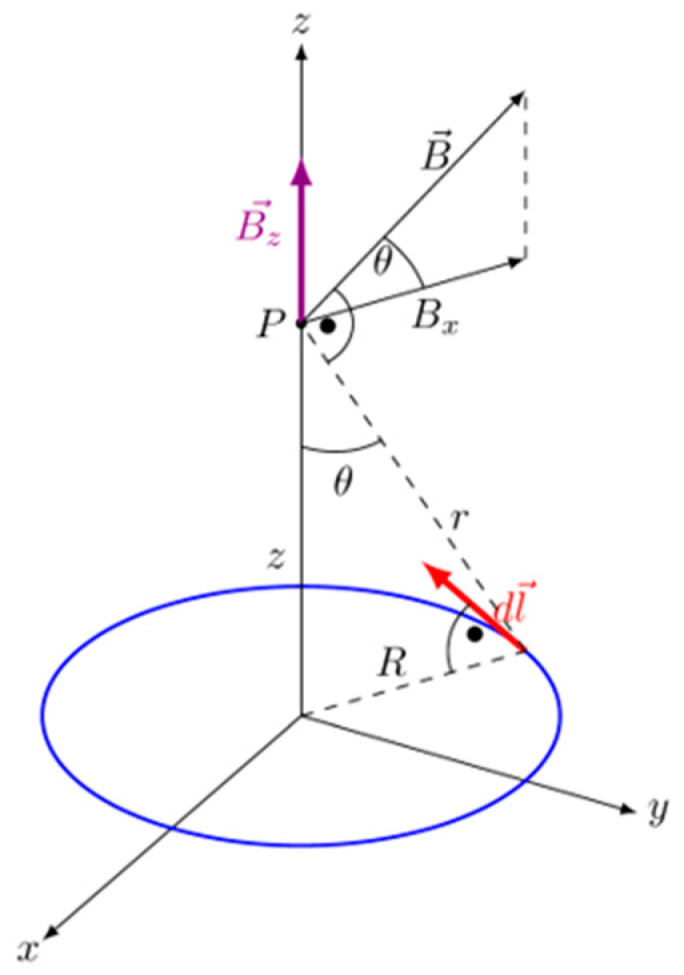
Illustration for the Biot–Savart law: singular loop of conductor.

The geometrical scheme for the singular loop of the conductor and the used designations are shown in Figure 4. Considering the symmetry of the system and analyzing point P on the symmetry axis of the winding, Equation (1) can be simplified into the form describing axial component of the magnetic field induction, Equation (2):

(2) $$\frac{dB_{z}}{\sin\theta }=\frac{\mu_{0}}{4\pi }\frac{IRd\vec{l}}{{\left(R^{2}+z^{2}\right)}^{\frac{3}{2}}}$$

After integrating around the full loop circumference, Equation (2) can be presented in its final form (Equation (3)). It is the classic form describing magnetic induction in singular coil. In the case of multilayer magnetic coils this result can be generalized by summarizing windings and layers (Equation (4)).

(3) $$B_{z}=\frac{\mu_{0}}{2}\frac{IR^{2}}{{\left(R^{2}+z^{2}\right)}^{\frac{3}{2}}}\;$$

(4) $$\vec{B_{sol}}=\frac{\mu_{0}I}{2}\sum_{k=0}^{K-1}\sum_{y=-\frac{L}{2d}}^{\frac{L}{2d}}\frac{{\left(R_{c}+kd\right)}^{2}}{{\left({\left(R_{c}+kd\right)}^{2}+{\left(yd\right)}^{2}\right)}^{\frac{3}{2}}}$$

where

*R*_c_—internal radius of the electromagnetic coil usable area; *L*—coil length (along the Z axis); *d*—thickness of singular layer (thickness of the wire) and *K*—number of coil layers.

Equation (4) does not account for the limitations resulting from the finite length of the coil and boundary conditions. The numerical model in Ansys Maxwell software version 2024 R2, enabling determination of spatial distribution of magnetic field induction, was prepared to overcome those limitations.

**Figure 5:**
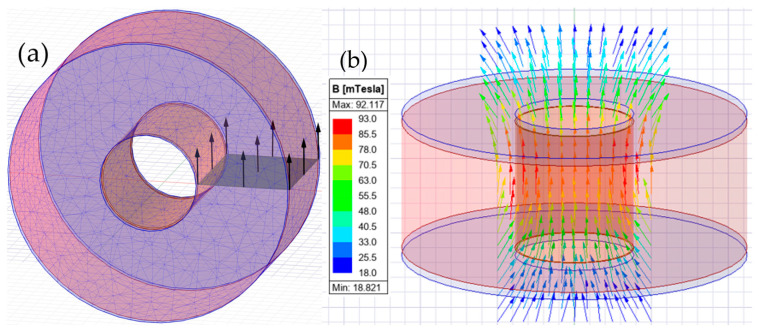
Numerical model representation of the electromagnetic coil in Ansys Maxwell software; (**a**) geometry with finite element mesh and electric current intensity vector, (**b**) magnetic field simulation results–distribution of the magnetic field induction vectors and graphical representation of field intensity.

The winding and air chamber geometry were determined (Figure 5). The material for the case was assumed to be non-magnetic. The finite element mesh size was assumed as 5 mm, see Figure 5a. The mesh was chosen experimentally considering the accuracy and time of the calculations.

Magnetic field calculations were performed under conditions reflecting real power supply of the electromagnet. Figure 5b presents different values of induction along the coil length and in its edge areas. The visible distribution confirms the presence of boundary effects, observed also in experimental measurements.

**Figure 6:**
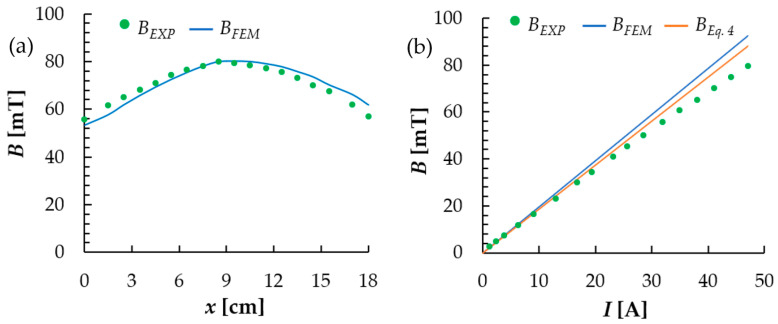
Validation of the magnetic field induction model: (**a**) axial profile of the induction along the coil axis—*B*(*x*); (**b**) *B*(*I*) dependency in the point of intersection of the symmetry axes (description in text).

To assess the adequacy of the model, the distribution of the magnetic induction along the coil axis (*x*, mm) is shown in Figure 6a. Experimental measurements of *B_EXP_* are presented as points, while numerical results *B_FEM_* are presented as a continuous line. The profile has one maximum in the middle part (*x* ≈ 8.5 cm) with convergent extremal values: 80 mT for *B_EXP_* and 80.2 mT for *B_FEM_*. The quantitative compatibility of the results was assessed based on the error values: mean absolute error *MAE* = 2.30 mT, mean absolute percentage error *MAPE* = 3.46%, root mean square error *RMSE* = 2.63 mT. The calculated value of the Pearson correlation coefficient equal to *r* = 0.95 demonstrates high resemblance of both curves. Highest noted difference in terms of *B_EXP_* and *B_FEM_* results is equal to 4.7 mT (about 8.2% in reference to measured value), located around *x* ≈ 18 cm, which can be caused by boundary effects, accumulation of the probe positioning inaccuracies (Hall-effect sensor used to measure the *B* values) and the model discretization. Overall, the model accurately represents the location and value of the maximum, as well as the curve shape, while the deviations grow getting closer to the boundaries of analyzed part.

The validation of magnetic induction *B* as a function of electric current intensity *I* in the point of intersection of the symmetry axes is presented in Figure 6b. Experimental results are presented along with analytical model predictions (as per Equation (4)) and FEM calculation results. In each case, *B-I* dependency is linear. Slope values for those linear functions are as follows: 1.75 mT/A for the measurements, 1.87 mT/A for analytical considerations and 1.96 mT/A for FEM model. Values calculated analytically and using FEM are overestimated in reference to the measurements’ result by 6.8% and 11.9%, respectively. The quantitative compatibility of the results was also assessed based on the error values, which are the following: *MAE* = 2.04 mT, *MAPE* = 6.56% and *RMSE* = 2.68 mT for analytical model and *MAE* = 3.58 mT, *MAPE* = 9.9% and *RMSE* = 4.59 mT for FEM. The source of the observed differences can be seen in the geometric deviation between real and modeled electromagnet dimensions (diameter and coil length assumed in calculations).

Based on the above presented analysis, the literature data and our own experimental observations, the magnetic induction value was chosen. Nováková et al. [48] noted that the magnetic field in the range from several dozen to several hundred mT is needed for the reorientation of short steel fibers. Mu et al. [35], on the other hand, confirmed the high efficiency of the orientation using comparable electromagnetic arrangements. In this study, 80 mT was chosen as an optimal value of the magnetic induction, ensuring sufficient effectiveness of fiber orientation and simultaneously providing stability of the mixture, based on the comparison between experimental and numerical results from ANSYS software. The experimental investigation was carried out in the model environment (gel, KONIX, Warsaw, Poland, with the same consistency as mortar; spread diameter of 20 cm) to preliminary asses the effectiveness of fiber orientation. A similar model medium was used for the preliminary assessment of 8 mm long fibers orientation in a magnetic field by Al Rifai et al. [39], while Wijffels et al. [29] observed visual effects of the magnetic orientation of singular and multiple fibers in a different medium—engine oil.

**Figure 7:**
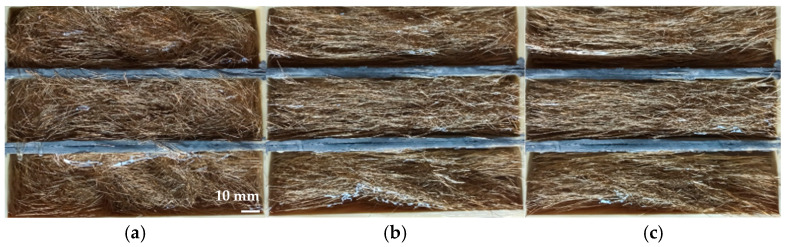
Preliminary investigations on the influence of magnetic field and vibrations on steel fiber orientation performed in model environment: (**a**) randomly oriented fibers (without magnetic field), (**b**) fibers submitted to the influence of magnetic field with induction of 80 mT and (**c**) fibers submitted to the influence of magnetic field with induction of 80 mT and mechanical vibrations (own photos).

In the preliminary research, fibers in the amount of *V_f_* = 2% were introduced into the gel and were submitted to magnetic field influence (80 mT) with and without vibration (Figure 7). Visual observations revealed significant differences in fiber arrangement between the specimens prepared: without the magnetic field, with magnetic field but without vibrations and with the combined influence of magnetic field and vibrations. The preliminary investigations confirmed that the highest efficiency of fiber orientation was achieved for the simultaneous use of a magnetic field and mechanical vibrations, which confirmed the validity of the adopted parameters.

To provide synchronization and accurate acquisition of data from various sources (MG-3002 sensor, temperature sensors (ABC-RC, Kraków, Poland), and clamp-on ammeter (UNI-T, Dongguan, China)), an original script in Python language (Python 3.10 (Python Software Foundation, Beaverton, OR, USA, https://www.python.org)) with graphical user interface (GUI) was created. The program enabled the choice of communication ports, the coil exploitation control, data export in CSV format and real-time preview. Data stream integration guaranteed the integrity of experimental results. The system enabled simultaneous monitoring and archiving of the results.

### 2.2. Experimental Methodology

#### 2.2.1. Digital Image Correlation (DIC) Settings

Displacement and strain registration using the Digital Image Correlation (DIC) method was performed utilizing the Aramis SRX (Zeiss, Oberkochen, Germany) system equipped with two camera heads, resolution of 2 × 12 MP (4096 × 3068 px). The cameras were positioned at a distance of 70 cm from the specimens, with 300 mm focal length lenses, which enabled achieving the measurement volume of 170 mm × 165 mm × 125 mm. The specimens’ surfaces were prepared by applying a speckle pattern, which was achieved by spraying the white background with black spots. The quality of the pattern was assessed each time using the Aramis SRX software version 2024. The pattern quality coefficient was, in each case, not lower than 4+, which in this measurement system is considered an acceptable level in terms of ensuring the correctness of the correlation. The LED lamp was positioned in such manner that the reflects and shadows were eliminated to achieve even lightning. The correlation parameters were identical for all the mechanical property tests and were as follows: 21 px—facet size and 16 px—facet step. The parameters were chosen considering the stability of the correlation (proper count of spots in each facet) and the density of the measurement mesh (high spatial resolution in strain concentration areas). Assuming the above-mentioned measurement geometry, those parameters corresponded to 0.9 mm of analyzed area for singular facet and 0.66 mm translation between following measurement points. Those parameters are in the range of the values presented in the literature discussing the use of DIC for cementitious composites [49]. The maximum allowable facet deviation equal to 0.3 (dimensionless) was assumed as the fit efficiency criterion. The frequencies of image acquisition were equal to 1 Hz and 5 Hz for specimens without and with fibers, respectively, which enabled the accurate capture of the initiation and propagation cracking processes. The image registration was synchronized with force and time recordings from the press. The force signal was submitted to preliminary median filtering to reduce the influence of single disturbances.

#### 2.2.2. Mechanical Properties

##### Flexural Performance Test

**Figure 8:**
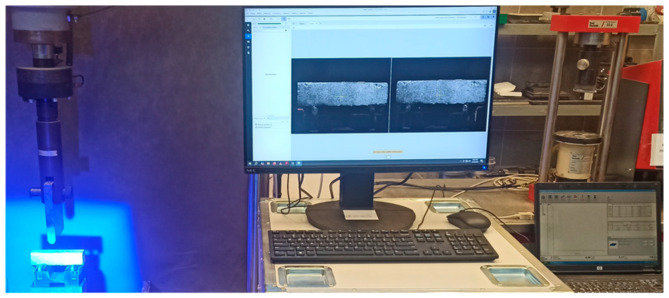
Research stand for the three-point bending (3PB) tests, enabling strain field registration with the use of DIC method: camera and lighting (**left**) and stand for image and force acquisition/analysis (**right**).

All the beams were subjected to the three-point bending (3PB) tests after 28 days from casting, using the ToniNORM press (Toni Technik, Berlin, Germany, range from 0 to 100 kN), in a setting with support span equal to 100 mm. The specimens were supported on two identical steel cylinders with the Ø10 mm diameter, while the load was transferred in the middle of the span through Ø16 mm steel roller (Figure 8). The tests were carried out in displacement-control manner with a loading rate of 0.35 mm/min.

The tests were performed in line with the EN 196-1 [46] standard recommendations with the following modifications: the displacement controlled loading and the use of DIC to register the beams’ displacements and strain fields (Figure 8). The deflection measurements in the middle of the span were adjusted by subtracting averaged settlements of the supports, measured by the means of DIC. In the whole loading range, the settlement values were approximately constant and equal to about 0.02 mm. Based on the registered force-deflection (*F-δ*) dependencies, the following properties were analyzed: flexural tensile strength *f_ct, fl_*, flexural elastic modulus *E_f_* (Equation (5)), ultimate deflection *δ_u_* corresponding to the maximum force (*F_max_*), work of fracture *W_f_* (Equation (6)), modified work of fracture W_f_* (Equation (7)) and the force corresponding to the assumed value of deflection δ* in the post-peak range, designated as *F*(δ*). The limiting deflection value assumed as 3 mm was based on the concept presented in the EN 14651 standard [50], while simultaneously enabled the comparability of the results between series (integration of *F-δ* curve up to same deflection value).

(5) $$E_{f}=\frac{\Delta F}{\Delta \delta }\left(\frac{L^{3}}{4bh^{3}}+\frac{L(1+\nu )}{2bh\kappa }\right)$$

where

*L*—beam span; *b* and *h*—dimensions of the beam’s cross section; Δ*F* and Δ*δ*—force increment (0.8–1.3 kN) and corresponding deflection increment; *ν*—Poisson ratio; *κ*—shape factor equal to 5/6 for rectangular cross section; the equation arises from the Timoszenko model, which is a modification of the classical Euler–Bernoulli theory, involving the shear deformations. For the small slenderness (*L/h*) values (2.5 for the specimens analyzed herein), shear participation significantly increases the calculated *E_f_*; therefore, the necessity of this adjustment. *ν* = 0.20 was assumed as the typical value for cement mortars [51].

(6) $$W_{f}=\frac{1}{V}\int_{0}^{\delta_{0.2}}F\left(\delta \right)d\delta ,\;\delta_{0.2}\;:\;F\left(\delta_{0.2}\right)=0.2F_{max}\;$$

(7) $$W_{f}^{*}=\frac{1}{V}\int_{0}^{\delta^{*}}F\left(\delta \right)d\delta ,\;\delta^{*}=3\mathrm{mm}$$

where

*V*—beam’s volume.

##### Compression Test

**Figure 9:**
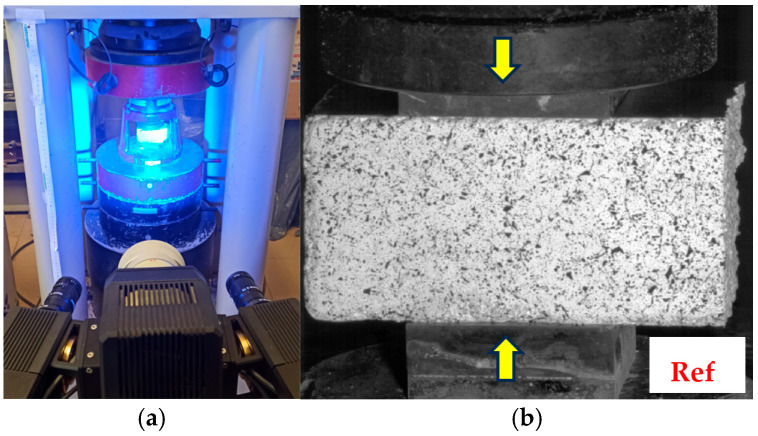
Research stand for compressive strength tests of beams’ halves and DIC measurement area: (**a**) stand with pressure plates, camera and lighting; arrow means the direction of loading; (**b**) specimen with prepared measuring surface (speckle pattern) mounted in the press before compressive strength test (own photos).

Halves of the beams obtained from the tensile tests were then submitted to compressive strength tests as per the [46] standard. The tests were performed on the ToniNORM press (Toni Technik, Berlin, Germany) with a range of up to 3000 kN. Pressure plates 40 mm × 40 mm were used (Figure 9), enabling the uniform transfer of loads to the specimen surface. Analogically to the tensile strength tests, the specimens were loaded at the rate of 0.35 mm/min, and strain maps were registered with the use of DIC.

#### 2.2.3. Microstructure

**Figure 10:**
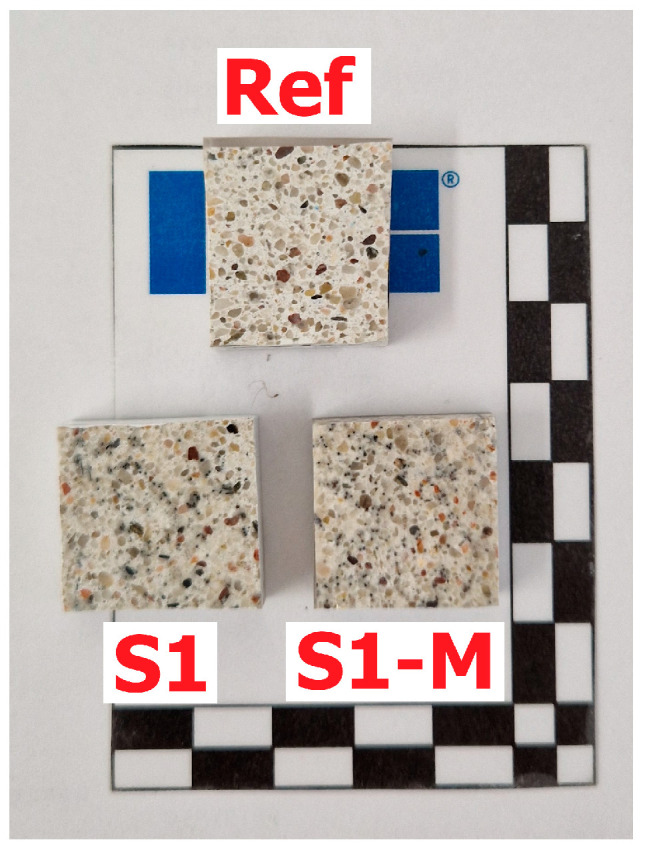
Polished section of samples prepared for microstructural investigations.

The microstructural analysis of the Ref, S1 and S1-M samples was carried out on smaller pieces of mortars after the bending test, cut perpendicular to the troweling surface at a distance from a damaged zone. Investigated surface area had dimensions of about 20 mm × 20 mm. After drying in the oven in 40 °C, the samples were immersed in resin under vacuum. In the next step, they were grinded and polished to receive smooth surface. Details of the sample preparation are described in one of the previous publications [52]. The samples prepared for analysis are presented in Figure 10.

Microstructural analysis was carried out in two steps—first using optical microscopy and second using scanning electron microscopy (SEM). Investigations in reflected visible light were made using stereoscopic microscope model Stemi 508 manufactured by ZEISS (Carl Zeiss Microscopy GmbH, Köln, Germany). After gold evaporation, the samples were investigated using SEM model Sigma 500 VP manufactured by ZEISS (Carl Zeiss Microscopy GmbH, Köln, Germany). Backscattered electron images (BSE) were collected. Analysis of elemental composition and maps of elements were made using energy dispersive X-ray detector (EDX) model Ultim Max 40 manufactured by Oxford Instruments (Oxford Instruments, High Wycombe, UK).

The aim of the microstructural analysis was to discover differences in the microstructure of the mortar containing steel fibers and fibers with ordered orientation referring to the reference sample without fibers. Spotted differences in the microstructure might help to explain differences observed in mechanical properties of cement composites. The nature of the observations carried out was qualitative rather than quantitative.

## 3. Results and Discussion

### 3.1. Force–Deflection Curves

**Figure 11:**
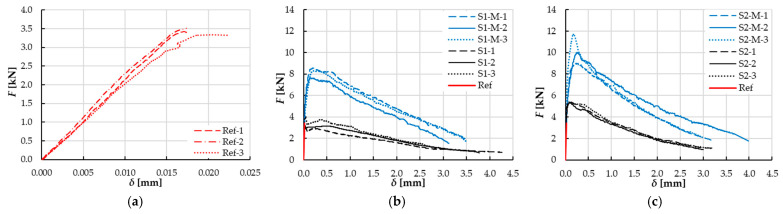
Force–deflection curves for beams: (**a**) reference specimens (without fibers), (**b**) specimens with fibers, *V_f_* = 1% and (**c**) specimens with fibers, *V_f_* = 2%; S—randomly oriented fibers, S-M—magnetically oriented fibers.

**Figure 12:**
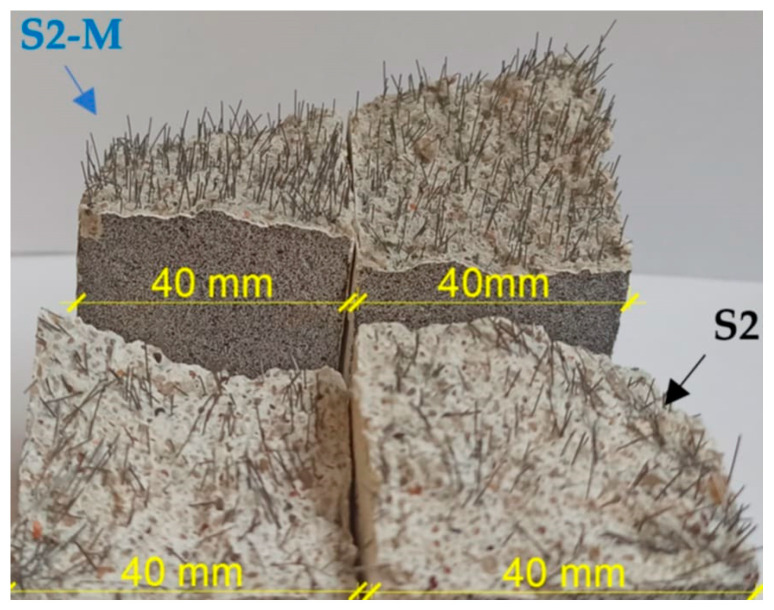
Cross sections of specimens with randomly (S) and magnetically (S-M) oriented fibers (own photo, not to scale).

Figure 11 illustrates the force–deflection (*F–δ*) curves for the beams. The course of the *F–δ* dependencies for reference specimens (without fibers) was linear, up to the fracture moment, after which rapid force reduction was noted, without the ability to carry out load along with further deflection increase (Figure 11a). In the case of fiber reinforced specimens, the bridging effect was observed, enabling carrying out loads by fibers in the cracked cross-sectional area in the postcritical stage (Figure 11b,c). However, for the specimens with 1% vol. randomly oriented fibers (S1 series), the maximum force *F_max_* was comparable to the value for the reference specimens (Figure 11b), while for 2% (S2) increase by about 58% was noted, when compared to the reference series. The reason for that was that the amount of bridging fibers in crack initiation cross section might have been too small to significantly increase the value of the maximum load for S1 series (Figure 12) due to, amongst other factors, their random arrangement [26,53].

For the specimens with the same content of steel fibers, higher mean values of maximum strength *F_ma_*_x_, as well as higher values of force corresponding to limiting deflection *δ* = 3 mm − *F*(δ*), were noted for the series with magnetically oriented (S-M) fibers when compared to those with random arrangement (S). The residual-to-peak ratio at a set deflection was higher for composites with magnetically oriented fibers than for those with random orientation. The values were equal to 0.31 for S1-M series, 0.29 for S1, 0.24 for S2-M and 0.20 for S2.

For the series with 1% vol. dosage, the increase by 133% and 148% was noted for *F_ma_*_x_ and *F*(δ*), respectively, while for 2%, it was 90% and 130%. The observed trend of lower increase of *F_max_* for higher fiber dosage is in line with observations made by Da Silva Brito et al. [26], who were investigating composites based on CEM 52.5R cement, silica fume, metakaolin and quartz sand with steel fibers (*λ* = 12.5/0.5). In their research, the composites with magnetically oriented fibers, compared to those with randomly oriented fibers, demonstrated higher pace of force decrease in postcritical stage and increase in maximum force value by ~72% and 22.6% for *V_f_* = 1% and 2%, respectively. The differences may result from the use of fibers of lower slenderness *λ* = 12.5/0.5 (*V_f_* = 1–5%) in comparison to this study (λ = 13/0.2). However, the absolute percentage difference in terms of *F_max_* increase (observed for the specimens with magnetically oriented fibers in reference to random arrangement) between the series with *V_f_* = 1% and 2% steel fibers dosage was equal to 49.6% and was similar to the value in the current study: 43%. Fernandez et al. [32] noted a 10% increase in *F_ma_*_x_ value for composites reinforced with steel fibers (λ = 22/0.5) in amount of 1%. In this case, however, the composites were prepared with the use of a recycling aggregate.

*F_ma_*_x_ values increased for specimens with higher fiber content in reference to those with smaller fiber content by 54% in the case of random fiber orientation and by 26% for magnetically oriented fibers (Figure 11b,c).

The F–δ curves slope after achieving F_max_ value was significantly steeper for specimens with magnetically oriented fibers (S-M) than for those with random arrangement (S) (Figure 11b,c). However, the profiles of the F–δ curves for the specimens with both randomly and magnetically oriented fibers are similar, which is in line with earlier investigations [27,37,40].

### 3.2. Mechanical Properties

Table 3 and Table 4 summarize the results from the mechanical properties tests. Table 3 presents the results from the flexural performance tests carried out on three beams, and Table 4 presents the results from the compressive strength (*f_c_*) tests. To assess whether a sample size of *n* = 3 was sufficient to reliably demonstrate differences, a one-way analysis of variance (ANOVA) was performed. Pairwise comparisons were also performed using Welch’s test, at a significance level of *α* = 5% with Holm’s correction. Effect sizes were assessed using the *η*^2^ coefficient for ANOVA and Hedges’ g coefficient for pairwise comparisons. The F statistic represents the ratio of between-group to within-group variability, and the *p*-value indicates the probability of obtaining observed differences in the absence of a true effect. A power analysis was also performed to determine the minimum sample size required to achieve a power of at least 0.80. ANOVA confirmed significant differences for the analyzed composite properties: *f_ct,_ _fl_* (*F* = 58.15, *p* < 0.001, *η*^2^ = 0.96); *δ_u_* (*F* = 54.57, *p* < 0.001, *η*^2^ = 0.96); *W_f_* (*F* = 73.34; *p* < 0.001; *η*^2^ = 0.97); W_f_* (*F* = 79.23, *p* < 0.001, *η*^2^ = 0.97) and *F*(δ*) (*F* = 9.77, *p* = 0.005, *η*^2^ = 0.79). The smallest significant pairwise effects were significant: *f_ct,_ _fl_ g* ≈ 3.98 (S2 vs. S2-M), *δ_u_ g* ≈ 2.13 (S1 vs. S1-M), *W_f_ g* ≈ 2.00 (S1 vs. S2) and W_f_* g ≈ 2.77 (S1 vs. S2). With *n* = 3, the power of the ANOVA test was close to ~1.00. For the flexural modulus (*E_f_*), one-way ANOVA was insignificant (*F* = 1.27; *p* = 0.34; *η*^2^ = 0.34). The differences related to fiber orientation in the magnetic field were small (S1 vs. S1-M: *g* ≈ 0.06; S2 vs. S2-M: *g* ≈ 0.68), while the effect of the fiber content with random orientation was moderate (S1 vs. S2: *g* ≈ 1.07). For *F*(δ*), the comparison of random and magnetic fiber orientation (S1 vs. S1-M) gives a very large effect (*g* ≈ 2.94), so *n* = 3 is sufficient.

**Figure 13:**
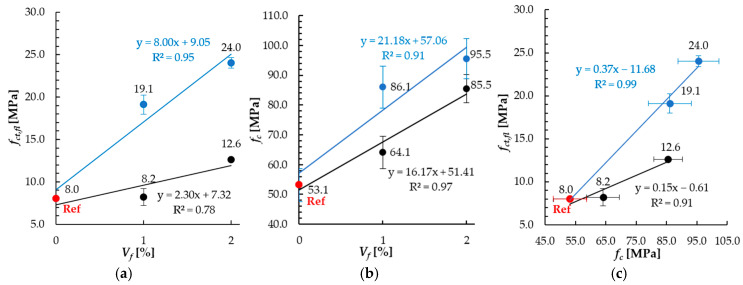
Relations: (**a**) *f_ct, fl_*-*V_f_*, (**b**) *f_c_*-*V_f_* and (**c**) *f_ct, fl_*-*f_c_* for mortars with randomly (black line, S series) and magnetically (blue line, S-M series) oriented fibers.

In each case, the mean value ($\bar{x}$), the standard deviation (*s*) and the coefficient of variation (CV) were calculated. Some parameters demonstrate high coefficients of variation. The variation coefficient was equal to about 30% for the residual force results of magnetically oriented fibers. Such a high value may result from the differences in fiber orientation in reference to magnetic field lines and local differences in the Interfacial Transition Zone area, which influences the ability of fibers to bridge cracks. Therefore, conclusions regarding differences resulting from fiber content on the force *F*(δ*) were formulated conservatively and will be the subject of future research. The sensitivity of adopted measurement system could have the influence on variability of the results in terms of flexural elastic modulus (over 20% for magnetically oriented fibers), as deflection was measured in very low loading range Δ*F*, see Equation (5). All the analyzed mechanical properties improved with the increase in fiber content (Figure 13). The improvement of the flexural tensile strength *f_ct, fl_* was significantly higher for the specimens with magnetically oriented fibers (S-M) than for those with randomly oriented fibers (S), see Figure 13a. The 1% vol. fiber dosage resulted in the increase (in comparison to reference specimens) of flexural strength by 139.2% and 2.5% for magnetic and random fibers orientation, respectively, while for 2%, it was 200.4% and 57.9%.

Same as in the case of flexural strength, the compressive strength growth was much higher for the specimens with magnetically oriented fibers (S-M) than for those with random arrangement (S). However, the magnetic orientation effect is significantly weaker (Figure 13b). The increase in compressive strength resulting from magnetic orientation (S-M series compared to S) was noted as 34.3% and 11.7% for 1% vol. and 2% vol. fiber dosage, respectively. Additionally, partial overlapping of the error bars limited the potential for a clear-cut distinction between the results.

The flexural tensile strength (*f_ct, fl_*) increased with the compressive strength (*f_c_*) increase, see Figure 13c, which is in line with observations made by Javahershenas et al. [34]. In this study, the dependency between *f_ct, fl_* and *f_c_* was linear. Higher slope coefficient value can be observed for specimens with magnetically oriented fibers (equal to 0.37) than for those with random orientation (0.15). This means higher flexural strength increase per compressive strength unit in the considered range. figure

**Figure 14:**
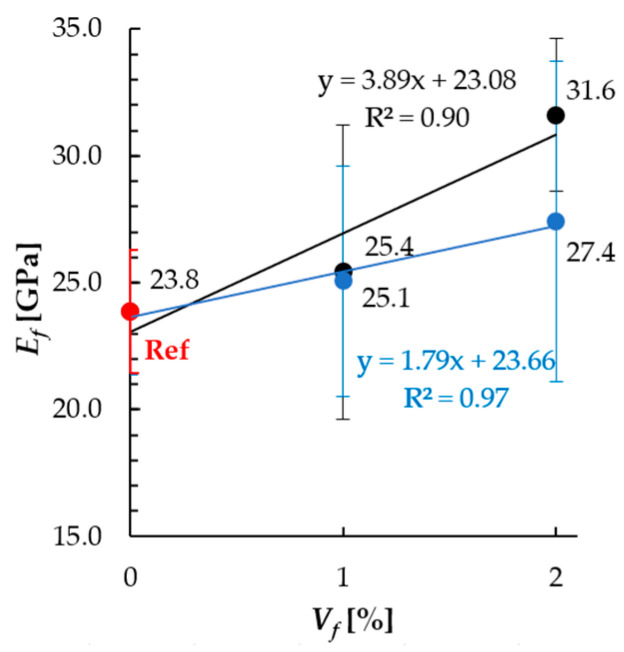
Relation between the flexural elastic modulus *E_f_* and fiber dosage *V_f_* for cement mortar with steel fibers oriented randomly (S series, black line) or magnetically (S-M series, blue line).

In terms of mean flexural elastic modulus *E_f_*, the values determined in this study were in the range from 23.8 GPa to 31.6 GPa. Also, similar values were noted for cementitious composites with steel fibers in the available literature data [29,54]. However, considering different specimens’ dimensions and differences in the calculation assumptions (e.g., Wijffels et al. used longer beams—100 mm × 100 mm × 400 mm, *L/h* ≈ 4 and Euler–Bernoulli model), direct comparison of the results is limited. The relation between flexural elastic modulus in tension *E_f_* and fibers content is presented in Figure 14. For the 1% vol. dosage, the increase in flexural elastic modulus by 6.6% and 5.2% in reference to non-reinforced specimens was noted for random and magnetic orientation, respectively. For 2% vol. dosage, it was 32.7% and 15.1%.

**Figure 15:**
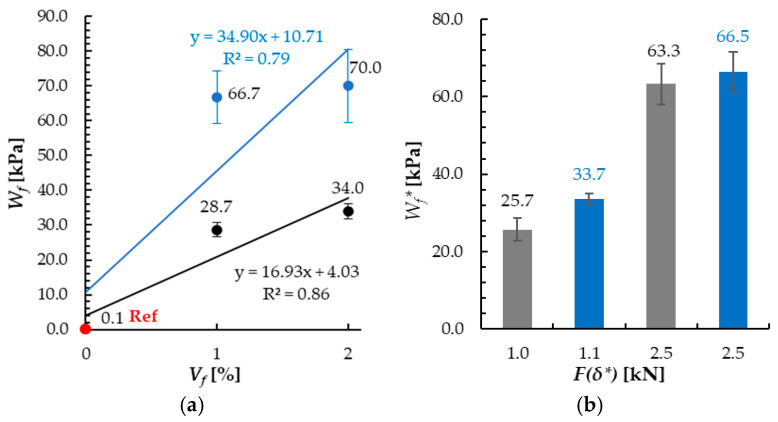
Energy absorption potential for cement mortar with fibers oriented randomly (S series, black line) or magnetically (S-M, blue line)—volume-normalized fracture work (in bending): (**a**) correlation between *W_f_* and *V_f_* and (**b**) correlation between W_f_* and *F*(δ*).

The fracture work (*W_f_*) increased with the fiber dosage (*V_f_*) growth, regardless of the fibers’ orientation method (Figure 15a). The increase was significantly higher for the specimens with magnetically oriented fibers in comparison to those with random arrangement and was equal to 34.9 kPa and 16.9 kPa for 1% vol. fiber dosage, respectively. For the 2% vol. dosage, the *W_f_* values in S-M series were more than twice as big as in the S series. The observed effects in terms of higher work of fracture (*W_f_*) values for higher fibers dosage and for magnetically oriented fibers are in line with the data available in the literature [27,37,39,55]. However, the calculated values cannot be directly compared due to the differences in the coefficients’ definitions and integration ranges.

In this study, higher *F*(δ*) values are accompanied by higher Wf* values (Figure 15b). In each case, the results were higher for magnetic oriented fibers than for those with random arrangement. The deflection values *δ_u_* for 1% vol. fibers dosage were equal to 211% (randomly oriented fibers) and 178% (magnetically oriented) of the values registered for reference, non-reinforced specimens. For 2% vol. fiber dosage, it was 162% and 195%, respectively.

**Figure 16:**
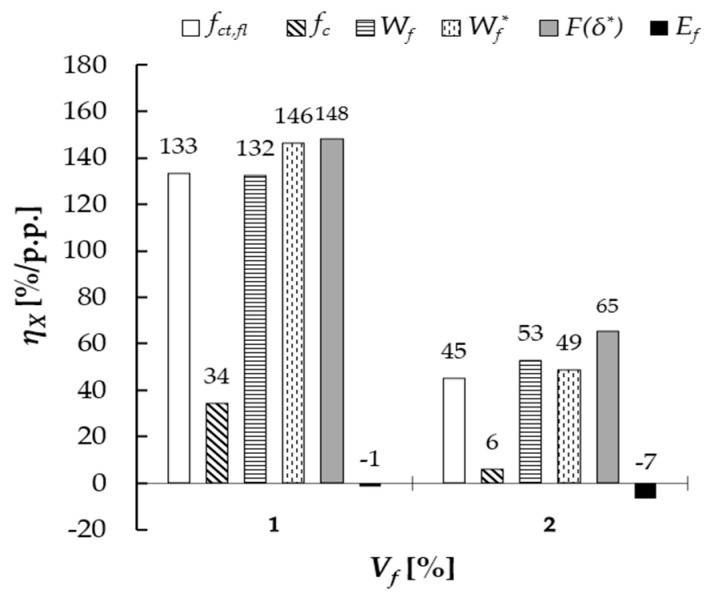
Efficiency factor *η_x_*—change in the analyzed property normalized by volumetric percentage of fibers content.

The efficiency factor *η_X_* was introduced to determine the influence of magnetic orientation of microfibers on the analyzed mechanical properties, defined as the relative change in the analyzed property for S-M series in comparison to S series referred to 1 percentage point of fibers vol. content (Equation (8)). Figure 16 presents the results of analysis. Higher values of *η_X_* for 1% vol. fiber content were noted for the residual force, fracture work and flexural strength: *F*(δ*) ≈ 148%/p.p, W_f_* ≈ 146%/p.p, *W_f_* ≈ 132%/p.p and *f_ct, fl_* ≈ 133%/p.p. For the compressive strength, the efficiency factor was equal to 34%/p.p., while for the flexural elastic modulus negative value was noted: −1%/p.p. Lower values were obtained in the case of 2% content, which were as follows: *F*(δ*) ≈ 65%/p.p., W_f_* ≈ 49%/p.p., *W_f_* ≈ 53%/p.p., *f_ct, fl_* ≈ 45%/p.p., *f_c_* ≈ 6%/p.p. and *E_f_* ≈ −7%/p.p. The positive influence of the magnetic orientation was therefore more noticeable for 1% fiber dosage than for 2%. The differences between the *η_X_* values corresponding to 2% and 1% were equal to *F*(δ*)—56%, W_f_*—67%, *W_f_*—60%, *f_ct,_ _fl_*—66% and *f_c_*—83%.

(8) $$\eta_{X}\left(V_{f}\right)=\left(\frac{X_{S-M}}{X_{S}}-1\right)\frac{100}{V_{f}[p.p.]}\;[\%/p.p.]$$

where

X_S-M_ and X_S_—are the mean values for magnetic and random orientation at the same fiber dosage *V_f_* (in percentage points).

It should be noted that during the preparation of the S-M series of composites, the magnetic field was applied simultaneously with mechanical vibrations. Therefore, the contribution of compaction cannot be fully separated from the effect of the magnetic field on fiber orientation. Furthermore, the mixtures from the reference series were not subjected to magnetic field induction. These conditions limit the interpretation of the results: the observed differences may result from both field-induced fiber orientation and densification effects, as well as the lack of field exposure of the reference samples.

### 3.3. DIC Analysis of Strain Localization in Bending (3PB) and Compression

**Figure 17:**
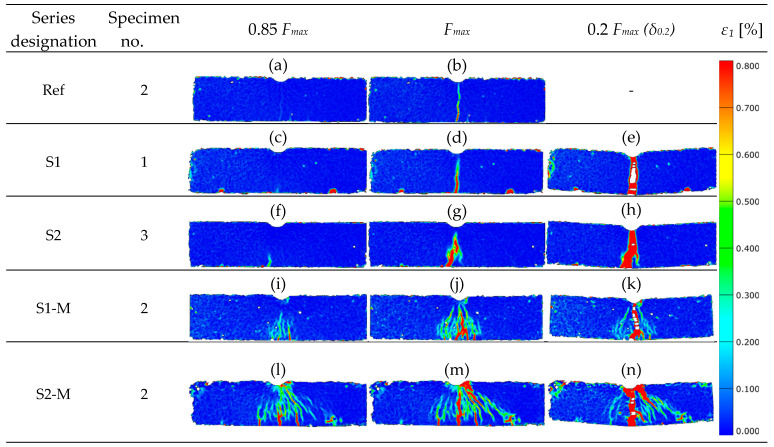
Principal tensile strain maps (*ε*_1_) for 3PB beams at load values 0.85 *F_max_*, *F_max_* and residual force 0.2 *F_max_* (*δ*_0.2_): (**a**) Ref for 0.85 *F_max_*, (**b**) Ref for *F_max_*, (**c**) S1 for 0.85 *F_max_*, (**d**) S1 for *F_max_*, (**e**) S1 for 0.2 *F_max_ (δ*_0.2_), (**f**) S2 for 0.85 *F_max_*, (**g**) S2 for *F_max_*, (**h**) S2 for 0.2 *F_max_ (δ*_0.2_), (**i**) S1-M for 0.85 *F_max_*, (**j**) S1-M for *F_max_*, (**k**) S1-M for 0.2 *F_max_* (*δ*_0.2_), (**l**) S2-M for 0.85 *F_max_*, (**m**) S2-M for *F_max_*, (**n**) S2-M for 0.2 *F_max_* (*δ*_0.2_).

The principal tensile strain maps *ε*_1_ [%] are shown in Figure 17 for beams submitted to three-point bending tests at three load levels: 0.85 *F_max_*, *F_max_* and residual force 0.2 *F_max_* (*δ*_0.2_). Strain localization bands (SLBs) were submitted to evaluation in terms of their count, orientation, *θ* (degree between the longer axis of SLB and vertical axis of the beam); deformation concentration *A_SLB_*/*A_ROI_* (*ROI*—region of interest) and *ε_1, peak_*, maximum *ε*_1_ value in the SLB. Only continuous bands of increased *ε*_1_ were considered for the SLB assessment. Singular red points, not connected to any band, were treated as so called “artifacts”, not influencing the SLB analysis.

A singular, thin band in the vertical direction was noted for the reference specimen (without fibers, Ref), see Figure 17b. Such cracking image is in line with the brittle failure mechanism (without post-peak stage on the *F-δ* curve, see Figure 11a). The strain values *ε*_1_ registered for *F_max_* force corresponded to the upper values of the scale, while SLB thickness was small (small *A_SLB_*/*A_ROI_* value). For the beams with fibers randomly oriented (S1 and S2, Figure 17d,g), the SLB area was broader in comparison to the reference beam (Ref), which resulted from the post-critical stage presence at *F-δ* curves (see Figure 11a,b). The thin-band scheme for SLB in the tensile zone was also noted by Michels and Gams [23] for randomly oriented fibers. However, in their study, normal tensile strains (*εₓ* instead of *ε*_1_) were analyzed, and a different scale/ROI was used.

A singular, vertical band (small *θ*) was dominant at the residual force corresponding to *δ*_0.2_ deflection for the beams with randomly oriented fibers; noticeable disruptions of the measurement continuity can be noted, which, however, did not influence the analysis results. In the case of the beam with 2% of fibers content, the SLB was noticeably broader (S2, Figure 17h) in comparison to the specimen with 1% vol. dosage. The *ε*_1_ strains in the SLB corresponded to the upper values of the scale at *F_max_*, while *A_SLB_*/*A_ROI_* deformation concentration factor was higher than for the reference beam (Ref).

In the case of beams with magnetically oriented fibers (S1-M, S2-M in Figure 17i–n) multipath, broader SLB areas were observed with skew bands at various angles, with higher *θ* values. It demonstrates that in the degradation process not only flexure but also shear was involved. The area contribution of the SLB in ROI (*A_SLB_*/*A_ROI_*) at *F_max_* was higher for the S-M type beams than for the Ref, S1 and S2 beams (see Figure 17j,m). The SLB area for beams with magnetically oriented fibers at the *δ*_0.2_ deflection was very spacious and took a significant part of the ROI (Figure 17k,n). Similar observations were made by Qing et al. [25] in their study focused on the axial tension of specimens with magnetically oriented fibers.

The fracture image captured for the specimens with magnetically oriented fibers was different than for those with random arrangement, for which the SLB areas were much tighter (Figure 17e,h). This observation is in line with higher values of *F_max_* and *W_f_* (Figure 11c) obtained for the S-M1 and SM-2 beams in comparison to Ref, S1 and S2 beams (Figure 11a,b).

**Figure 18:**
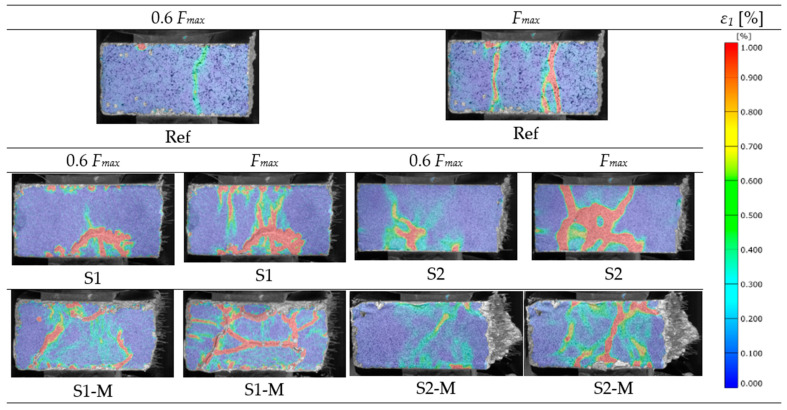
Principal tensile strain maps (*ε*_1_) for halves of the beams submitted to the compression tests at the load values of 0.6 *F_max_* and *F_max_*.

The principal strain *ε*_1_ maps for specimens submitted to compression are presented in Figure 18. The strain-localization bands were compared at loading levels of 0.6 *F_max_* and *F_max_* in terms of their count, continuity and orientation in reference to the loading direction (Figure 9b). In each series, *ε*_1_, *_peak_* increased between 0.6 *F_max_* and *F_max_*. Singular strain-localization band, parallel to the loading direction, was noted for the reference specimen (without fibers) at load level of 0.6 *F_max_*, while two bands, also parallel to the loading direction, were observed at *F_max_*.

Multiple, short SLBs, parallel to the loading direction and one longer, arched band, perpendicular to the loading direction, were noted in the specimen with 1% vol. content of randomly oriented fibers (S1) at the load level of 0.6 *F_max_*. At the same load level in the specimen with 2% vol. fiber dosage (S2), multiple short bands with low intensity were observed. At the *F_max_* loading level, the SLB areas in the S1 specimen got denser, joined one another and created irregular net without the dominant direction. In the S2 specimen, at the same load level, the SLB area in the multipath shape, with one centrally located band, was created.

For specimens with magnetically oriented fibers (S1-M, S2-M) for both analyzed load levels (0.6 *F_max_* and *F_max_*), the SLB areas with the net of branched, skew bands, not aligned with loading direction were observed. These fracture images differ from those observed for the other specimens (Ref, S1 and S2) by the presence of more spread brands of lower intensity.

### 3.4. Microstructural Observations

#### 3.4.1. Optical Microscopy Observations

**Figure 19:**
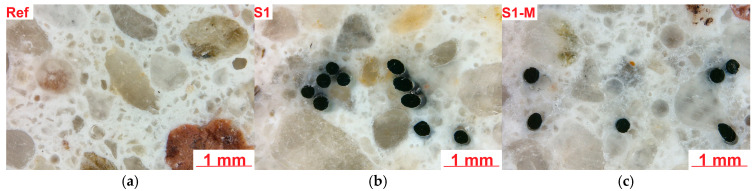
Microstructure of samples (optical microscopy): (**a**) Ref, (**b**) S1, (**c**) S1-M.

Figure 19 presents examples of images collected during the microscopic investigations of mortars using optical microscopy.

Microstructural analysis using optical microscopy of the Ref sample has shown grains of fine aggregate in the white cement matrix. The microstructure was dense and well compacted without excessive air voids. S1 and S1-M samples contained several steel fibers which were cut during sample preparation. Samples containing fibers were more porous, especially in the areas with agglomerates of fibers, which might be partly an effect of cutting the sample when the grout surrounding the fibers was destructed or an effect of air entrap during sample preparation. In the S1-M sample, most of the fibers were cut perpendicularly, which seems to be an effect of ordered magnetic orientation during molding.

#### 3.4.2. Scanning Electron Microscopy

**Figure 20:**
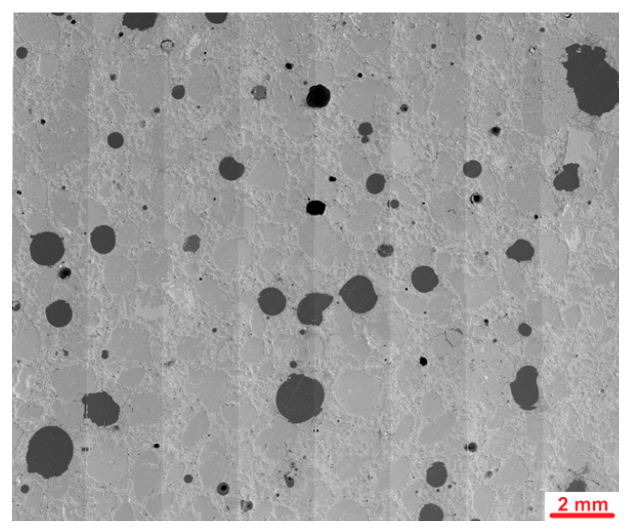
Stitched SEM images of the Ref sample.

**Figure 21:**
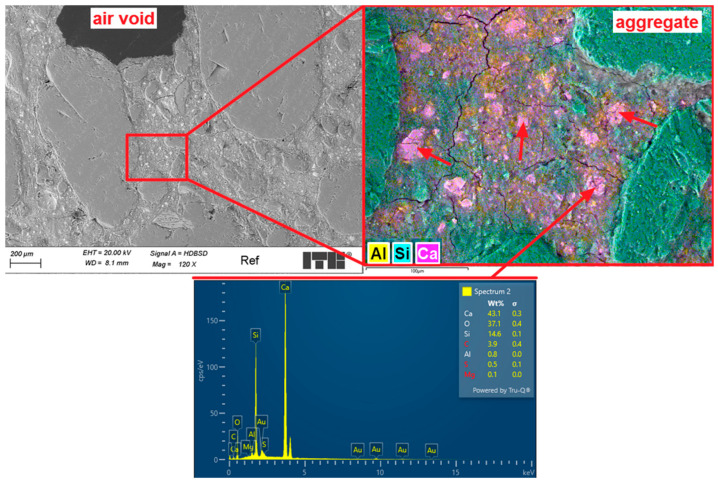
Microstructure of the Ref sample; arrows mark clinker relicts.

Figure 20 presents stitched SEM images of the investigated area of the Ref sample. Investigations of microstructure of the Ref sample have shown grains of fine aggregates mostly well covered by the cement matrix. Several macropores with dimensions up to 2 mm were observed. Most of them had spherical shape; however, their dimensions and distribution were far from those typically observed in frost resistant composites. Example of images collected during deeper investigations of microstructure is presented in Figure 21.

Analysis of the microstructure of the Ref sample showed that the fine aggregate contains mostly quartz grains. Cement matrix was tightly fitting to the surface of aggregate grains. The C-S-H phase was dense and well developed. Several relicts of white Portland cement clinker were observed. As a main difference between ordinary Portland cement clinker, white cement does not contain iron in its composition (Figure 21—EDX). Small dimensions of observed relicts demonstrate high rate of reaction, which is also caused by the high initial specific surface of used cement 52.5 R class. The observed microcracks in the cement grout were partially caused by the shrinkage of the cement matrix due to the high cement content, but they might also have shown up during sample preparation.

**Figure 22:**
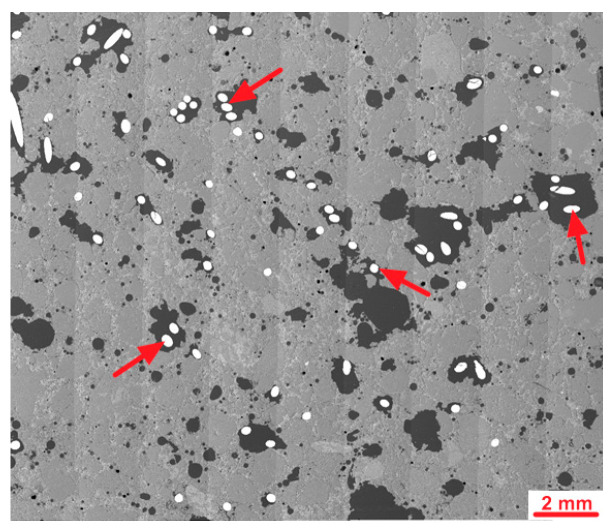
Stitched SEM images of the S1 sample; arrows mark steel fibers.

Figure 22 shows stitched SEM images of the investigated area of S1 sample.

**Figure 23:**
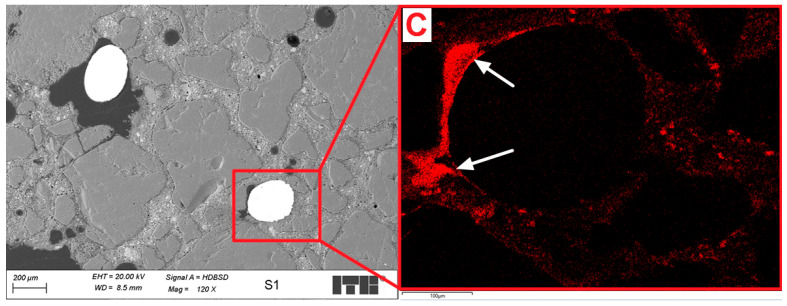
Microstructure of the S1 sample; arrows mark the discontinuous in the transition zone the on EDX carbon map.

**Figure 24:**
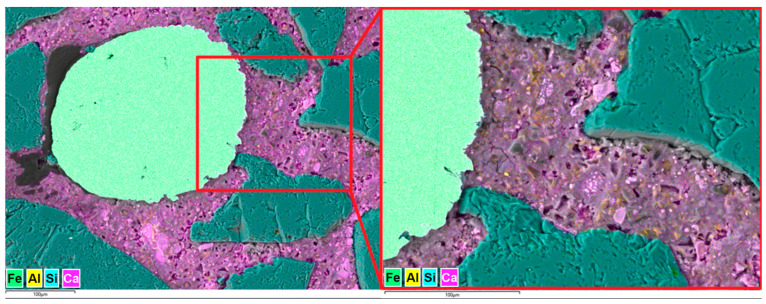
Microstructure of the S1 sample; area surrounding the steel fiber.

Analysis of the microstructure of the S1 sample compared to the Ref sample demonstrated some differences. First of all, several steel fibers were observed from which most were cut perpendicular; however, about 20% of the observed fibers were cut in other directions, showing ellipsoidal shape. Probably, if the size of the molded sample was bigger, the number of disordered fibers will also be higher. Second, the main difference was the highly increased area of air voids from which most had an irregular shape. Some of those air voids surrounding fiber agglomerates were maid probably during the cutting of sample as the remaining cement matrix in this area was cracked; however, others seem to be formed during the molding of the samples. Figure 23 and Figure 24 show examples of images collected during further investigations of the S1 sample microstructure.

The analysis of the microstructure of the S1 sample was focused mainly on the areas located near the steel fibers as the areas further located were similar to those observed in the Ref sample. Most of the observed steel fibers were more or less discontinuous in the transition zone between the cement grout and fiber. Figure 23 presents an example of such fiber. In the air void partially surrounding the fiber, resin was injected during sample preparation, and on the EDX carbon map, discontinuities are clearly visible in red color. Such discontinuousness will definitely cause a decrease in the summarized adhesion of steel fiber to the cement matrix and might decrease the mechanical properties of composite. Analyzing different areas of the same fiber (Figure 24), it might be seen that in this region, the transition zone between the fiber and the cement grout is sealed and tight. Additionally, the uneven surface of the steel fiber will increase the bonding, which is favorable due to the mechanical properties of the composite. During the observations of the cement grout in the S1 sample, less amount of microcracks were spotted, which might suggest that the addition of fibers also reduces shrinkage of the examinate composite.

**Figure 25:**
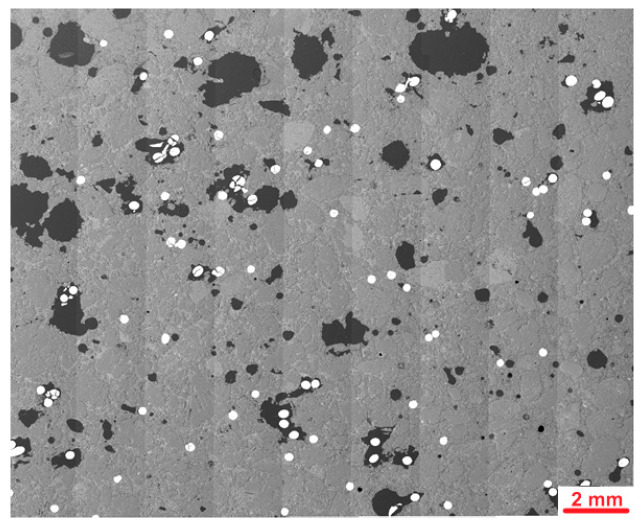
Stitched SEM images of the S1-M sample; arrows mark steel fibers.

**Figure 26:**
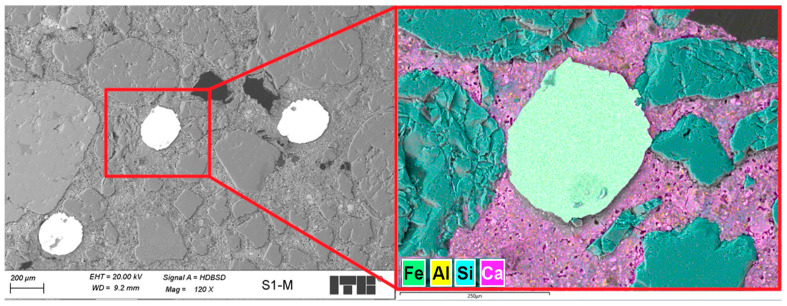
Microstructure of the S1-M sample; area surrounding a steel fiber.

Figure 25 presents stitched SEM images of the investigated area of the S1-M sample. Analysis of the microstructure of the S1-M sample also showed an increased number of air voids compared to the Ref sample; however, there were fewer of them compared to the S1 sample. All the observed fibers were cut perpendicularly, showing their clear circular surface, which proves their orderly distribution. A smaller amount of fiber aggregates was observed in the S1-M sample than in the S1 sample; however, no statistics were not carried out in this aspect, so it needs further investigations if a strong magnetic field is favorable for better distribution of the fibers. In the analyzed area of the S1-M sample, observed fibers were more frequently fully covered by the cement matrix than in the S1 sample. Figure 26 presents an example of images collected during further investigations of the microstructure of the S1-M sample.

Investigations of the microstructure of the S1-M sample in the area surrounding the steel fiber showed differences between those observed in the S1 sample. The main difference was that the cement matrix frequently fully surrounding the steel fiber, without any or with fewer discontinuities than in the S1 sample. The transition zone between the fiber and the cement grout was sealed and tight, which is a cause of the observed increase in mechanical properties. The magnetic field, apart from helping to orderly distribute steel fibers, might also help release some of the excessive air entrapped in the cement composited during sample preparation.

## 4. Conclusions

Based on the carried-out analyses concerning cementitious composites reinforced with microfibers oriented randomly or magnetically, submitted to bending and compression, the following conclusions were formulated:

- Maximum force (*F_max_*) values for the specimens with magnetically oriented fibers were higher by 133% (for *V_f_* = 1%) and by 90% (*V_f_* = 2%) than the values obtained for the specimens with random fiber arrangement, while fracture work *W_f_* values increased by 132% and 106%, respectively.
- All the analyzed mechanical properties increased with the fiber content growth. For 1% vol. dosage, increase in flexural strength by about 3% and 139% was noted in comparison to reference, non-reinforced specimens for randomly and magnetically oriented fibers, respectively. For 2% vol. dosage, it was 58% and 200%.
- For 1% vol. dosage, the increase in compressive strength by 21% and 62% was noted for specimens with random and magnetic orientation of fibers, respectively. For 2% vol. fiber dosage, it was 61% and 80%.
- Higher values of strength were noted for specimens prepared with the use of magnetic field. Increase in the flexural strength was equal to 133% and 90% for 1% and 2% fiber dosage, respectively, when comparing specimens with magnetically oriented fibers (S-M) to those with random arrangement (S). In the case of compressive strength, it was about 34% and 12%.
- Based on the efficiency factor (*η_X_*) values (Figure 10), the positive influence of fibers’ magnetic orientation was confirmed. The highest influence was demonstrated for the residual force value *F*(δ*), fracture work (W_f_*) and flexural tensile strength (*f_ct, fl_*); moderate for compressive strength (*f_c_*) and marginal for the effective elastic modulus (*E_f_*). The magnetic orientation beneficial effect was higher for *V_f_* = 1% than for *V_f_* = 2%.
- The use of magnetic field for the orientation of fibers changed the strain fields, as confirmed in the analysis of the *ε*_1_ principal tensile strain map created with the DIC method use in the flexural performance test. Multiple, skew bands were registered for the S-M type specimens instead of thin, singular bands noted for other beams. Singular (one or two) bands, parallel to the loading direction, dominated in the case of the reference specimens (without fibers) submitted to compression tests, while a net of wide-spread, skew (in reference to loading direction) bands was noted for specimens with magnetically oriented fibers.
- The proposed numerical model of the electromagnet performance was validated. The mean absolute percentage error (MAPE) was equal to 3.46%. A broader scope of investigation into the influence of different values of magnetic field induction and exposure time on fiber orientation in composites will be the subject of future research.
- Microstructural investigations have shown that the addition of steel fibers increases the air content in cement mortar, increasing the number of macropores, which is unfavorable. However, the magnetic field, apart from helping to orderly distribute steel fibers, seems to help release some of the excessive air entrapped in the cement composited during sample preparation. Microstructural analysis has shown that the magnetic field used during molding helps to fully cover the fibers with the cement matrix, which is beneficial for this type of cement composite and results in an increase in mechanical properties. It might also have an influence on decreasing the amount of fiber aggregates, although no statistics were carried out in this aspect, so it needs further investigations. Apart from the above, microstructural analysis has shown that the addition of steel fibers to cement composites reduces the number of microcracks, which are probably caused by a reduction in the shrinkage of the composite. The nature of the observations carried out was qualitative rather than quantitative. An in-depth quantitative analysis will be carried out as part of further research.

The results from this study demonstrate improvement in the mechanical properties of cementitious composite with magnetically oriented fibers in comparison to random orientation. Similar properties can be achieved with lower fiber dosage, which reduces steel usage and, as a result, carbon footprint. Additionally, fibers can be magnetically oriented along the trajectory of principal tensile stresses in prefabricated elements. This enables individual forming of dispersed reinforcement in various types of such elements to fulfill the requirements considering safety and serviceability of construction. The existing limitation is the necessity of preparing and introducing the proper technology using electromagnet. The combined influence of magnetic orientation of fibers and mechanical compaction of the fresh mix with fibers should be carefully analyzed to clearly assess the influence of magnetic orientation itself.

Further investigations should aim to involve the assessment of cementitious composites with fiber reinforcement of various structure and at higher dosage, as well as the verification of the results presented herein for hybrid fibers.

This article is the first in a publication cycle, which aims to introduce this technology to the prefabricated construction market.

## Figures and Tables

**Table 1:** Summary of the research investigations on steel fiber reinforced cementitious composites with magnetically oriented fibers.

Author		Year	Matrix	Fiber (typ, l/d)	*V_f_* [%]	*B* [mT]	*tB* [s]	Property	Effect
Michels and Gams	[28]	2016	M	H, 30/0.6	0.39–0.52	30	10	*f_ct, fl_*	~35
Wijffels et al.	[29]	2017	SCC	H, 50–60/0.8–1	0.5	20	30	*f_ct, fl_, G_f_*	~15–336
Abrishambaf et al.	[30]	2017	M	I, 9 i 12/0.175	1.5–3	-	-	*f_UTu_*	~82–91
Mu et al.	[20]	2017	M	I, 30/0.5	0.8–2	0.15	40	*f_ct, sp_, f_ct, fl_, T_150_*	~87–196
Abavisani et al.	[31]	2018	SCC	JSC, 15/-	0.9	500	-	*f_c_*	~17
Ferrandez et al.	[32]	2019	M *	I, 22/0.5	0.5–1	500	-	*f_c_, f_ct, fl_*	~10
Qing et al.	[25]	2019	M	I, 30/0.5	0.8–2	0.15	40	*f_UTu_, G_f_*	~26–57
Al-Ghalib and Ghailan	[21]	2020	SCC	H, 50/0.5 H, 32/0.4 I, 13/0.017	0.35–1.05	7.5	30	*f_ct, fl_*	~40
Hajforoush et al.	[33]	2021	C	H, 50/0.8	1–1.5	500	120	*f_c_, f_ct, sp_*	~7–18
Javahershenas et al.	[34]	2021	C	H, 50/0.8	0.4–1	500	60–180	*f_c_, f_ct, fl_*	~8–27
Da Silva Brito et al.	[26]	2021	M *	I, 12.5/0.5	1–5	9.5	40	*f_ct, fl_, f_c_, W_f_*	~22–115
Mu et al.	[35]	2021	M	H, I, 30/0.5	0.8–1.6	100	60	*f_ct, fl_*	~54–95
Ahmad et al.	[36]	2021	C	I, 8/0.2	0.8–2	0.15	40	*G_f_, K_I,C_*	36–108
Cao et al.	[27]	2022	C	I, 30/0.5	0.8–2	-	-	*G_f_, K_I,C_*	~30–170
Khan et al.	[37]	2022	M	H, 25/0.5	0.8–2	0.15	40	*f_ct, fl,_ f_R_, G_f_*	~51–86
Carrera et al.	[38]	2022	C	I, 13/0.15	1.5	100	10	*f_c_, f_ct, fl_*	9–179
Al Rifai et al.	[39]	2024	SCC	I, 8/0.2	0.7–1.05	10	60	*f_c_, f_ct, sp_, f_ct, fl_*	~1–29
Chen et al.	[40]	2024	M	H, 35/0.5	0.5–1.5	8	50	*f_ct, fl_, W_f_*	~19–46

Legend: M—cement mortar, C—concrete, SCC—self compacted concrete; H—hooked end fibers, I—straight fibers, JSC—jagged steel chips, *—dimensions of the specimens: 40 mm × 40 mm × 160 mm; *B*, *tB*—magnetic field induction, time of exposure, respectively; *f_c_*—compressive strength, *f_ct, fl_*—flexural tensile strength, *f_ct, sp_*—splitting tensile strength, *f_Utu_*—axial tensile strength, *f_r_*—residual stresses during bending, *G_f_*—fracture energy, *W_f_*—fracture work; *T_150_*—ductility index; *K_Ic_*—factor of stress intensity; effect—the highest increase in the analyzed property in reference to the specimens with random orientation of fibers [%].

**Table 2:** Mix compositions for each series (g).

Series	Cement	Sand	Water	Steel Fibers (*V_f_*)	Superplasticizer
Ref *	450	1350	225	0.0	0.0
S1				69.2 (1% vol.)	2.2
S2				138.4 (2% vol.)	3.0
S1-M				69.2 (1% vol.)	2.2
S2-M				138.4 (2% vol.)	3.0

* Reference sample.

**Table 3:** The results of the flexural performance tests (designations explained in the text).

Sample ID	*f_ct, fl_*	*E_f_*	*δ_u_*	*W_f_*	W_f_*	*F*(δ*)
	MPa	GPa	mm	kPa	kPa	kN
Ref-1	8.0	22.4	0.017	0.123	-	-
Ref-2	8.2	26.7	0.017	0.130	-	-
Ref-3	7.8	22.4	0.022	0.180	-	-
$\bar{x}$	**8.0**	**23.8**	**0.019**	**0.144**	-	-
***s***	**0.2**	**2.5**	**0.003**	**0.031**	-	-
***CV***	**2.7%**	**10.3%**	**15.1%**	**21.4%**	-	-
S1-1	7.9	23.4	4.26	26.7	22.6	0.98
S1-2	7.4	20.9	3.78	28.6	25.9	1.05
S1-3	9.3	32.0	3.97	30.8	28.5	1.04
$\bar{x}$	**8.2**	**25.4**	**4.01**	**28.7**	**25.7**	**1.02**
***s***	**1.0**	**5.8**	**0.24**	**2.0**	**2.9**	**0.04**
***CV***	**12.1%**	**22.8%**	**6.0%**	**7.1%**	**11.5%**	**4.0%**
S2-1	12.6	33.4	3.19	34.9	34.1	1.14
S2-2	12.5	28.1	2.86	31.6	32.1	0.95
S2-3	12.8	33.4	3.19	35.6	34.8	1.16
$\bar{x}$	**12.6**	**31.6**	**3.08**	**34.0**	**33.7**	**1.08**
***s***	**0.1**	**3.0**	**0.19**	**2.2**	**1.4**	**0.11**
***CV***	**1.0%**	**9.6%**	**6.3%**	**6.4%**	**4.1%**	**10.6%**
S1-M-1	20.1	30.0	3.52	72.4	67.5	2.91
S1-M-2	17.9	24.1	3.12	58.2	57.4	1.87
S1-M-3	19.4	21.1	3.50	69.5	64.9	2.84
$\bar{x}$	**19.1**	**25.1**	**3.38**	**66.7**	**63.3**	**2.54**
***s***	**1.1**	**4.5**	**0.23**	**7.5**	**5.2**	**0.58**
***CV***	**5.9%**	**18.1%**	**6.7%**	**11.2%**	**8.3%**	**22.9%**
S2-M-1	21.1	22.3	4.28	63.3	61.7	2.08
S2-M-2	23.5	25.5	3.99	82.0	71.9	3.34
S2-M-3	27.5	34.5	2.85	64.6	65.9	2.08
$\bar{x}$	**24.0**	**27.4**	**3.71**	**70.0**	**66.5**	**2.50**
***s***	**3.2**	**6.3**	**0.76**	**10.4**	**5.1**	**0.73**
***CV***	**13.5%**	**23.1%**	**20.4%**	**14.9%**	**7.7%**	**29.1%**

**Table 4:** The results of the compressive strength tests (MPa, designations explained in the text).

	Ref	S1	S2	S1-M	S2-M
	53.9	55.7	84.5	78.9	93.5
	54.6	62.1	77.9	89.1	94.5
	59.7	66.3	87.4	96.8	106.4
	44.6	67.5	90.5	82.7	87.8
	52.9	68.9	87.2	82.8	95.4
$\bar{x}$	**53.1**	**64.1**	**85.5**	**86.1**	**95.5**
***s***	**5.4**	**5.3**	**4.8**	**7.0**	**6.8**
***CV***	**10.3%**	**8.3%**	**5.6%**	**8.2%**	**7.1%**

## Data Availability

The original contributions presented in this study are included in the article. Further inquiries can be directed to the corresponding authors.

## References

[1] Furtak K. (2021). Contemporary challenges of science and technology-selected reflections. Cem. Wapno Beton.

[2] Czarnecki L., Gemert D. (2017). Innovation in Construction Materials Engineering versus Sustainable Development. Bull. Pol. Acad. Sci. Tech. Sci..

[3] Bywalski C., Kaźmierowski M., Kamiński M., Drzazga M. (2020). Material Analysis of Steel Fibre Reinforced High-Strength Concrete in Terms of Flexural Behaviour. Experimental and Numerical Investigation. Materials.

[4] Isa M., Pilakoutas K., Guadagnini M., Angelakopoulos H. (2020). Mechanical performance of affordable and eco-efficient ultra-high performance concrete (UHPC) containing recycled tyre steel fibres. Constr. Build. Mater..

[5] Sanjeev J., Nitesh K.S. (2020). Study on the effect of steel and glass fibers on fresh and hardened properties of vibrated concrete and self-compacting concrete. Mater. Today Proc..

[6] Małek M., Jackowski M., Łasica W., Kadela M. (2020). Characteristics of Recycled Polypropylene Fibers as an Addition to Concrete Fabrication Based on Portland Cement. Materials.

[7] Małek M., Jackowski M., Łasica W., Kadela M., Wachowski M. (2021). Mechanical and Material Properties of Mortar Reinforced with Glass Fiber: An Experimental Study. Materials.

[8] Faleschini F., Zanini M.A., Hofer L., Toska K., De Domenico D., Pellegrino C. (2020). Confinement of reinforced concrete columns with glass fiber reinforced cementitious matrix jackets. Eng. Struct..

[9] Khan Z.I., Arsad A., Mohamad Z., Habib U., Zaini M.A.A. (2020). Comparative study on the enhancement of thermo-mechanical properties of carbon fiber and glass fiber reinforced epoxy composites. Mater. Today Proc..

[10] Kossakowski P., Dygas J. (2023). Natural carbon fibres—An overview. J. Mater. Sci..

[11] Bayraktar O.Y., Yarar G., Benli A., Kaplan G., Gencel O., Sutcu M., Kozłowski M., Kadela M. (2023). Basalt fiber reinforced foam concrete with marble waste and calcium aluminate cement. Struct. Concr..

[12] Wydra M., Dolny P., Sadowski G., Fangrat J. (2021). Flexural Behaviour of Cementitious Mortars with the Addition of Basalt Fibres. Materials.

[13] Bencardino F., Mazzuca P., do Carmo R., Costa H., Curto R. (2024). Cement-Based Mortars with Waste Paper Sludge-Derived Cellulose Fibers for Building Applications. Fibers.

[14] Yoo D.-Y., Yoon Y.-S., Banthia N. (2015). Flexural Response of Steel-Fiber-Reinforced Concrete Beams: Effects of Strength, Fiber Content, and Strain-Rate. Cem. Concr. Compos..

[15] Olivito R.S., Zuccarello F.A. (2010). An Experimental Study on the Tensile Strength of Steel Fiber Reinforced Concrete. Compos. Part B Eng..

[16] Kadela M., Małek M., Jackowski M., Kunikowski M., Klimek A., Dudek D., Rośkowicz M. (2023). Recycling of Tire-Derived Fiber: The Contribution of Steel Cord on the Properties of Lightweight Concrete Based on Perlite Aggregate. Materials.

[17] Kaźmierowski M., Jaskulski R., Drzazga M., Nalepka M., Kordasz M. (2024). Effects of the Addition of Short Straight Steel Fibers on the Strength and Strains of High-Strength Concrete during Compression. Sci. Rep..

[18] Abdallah S., Rees D.W.A. (2019). Analysis of Pull-Out Behaviour of Straight and Hooked End Steel Fibres. Engineering.

[19] Lee C., Kim H. (2010). Orientation Factor and Number of Fibers at Failure Plane in Ring-Type Steel Fiber Reinforced Concrete. Cem. Concr. Res..

[20] Mu R., Li H., Qing L., Lin J., Zhao Q. (2017). Aligning Steel Fibers in Cement Mortar Using Electro-Magnetic Field. Constr. Build. Mater..

[21] Ghailan D.B., Al-Ghalib A.A. (2020). Magnetic Alignment of Steel Fibres in Self-Compacting Concrete. Aust. J. Struct. Eng..

[22] Soroushian P., Cha-Don L. (1999). Distribution and Orientation of Fibers Reinforced Conrete. ACI Mater. J..

[23] Miler A.I., Bjorklund F.R. (1977). Method of Reinforcing Concrete with Fibres. U.S. Patent.

[24] Svedberg B. (2004). Method and Device for Magnetic Alignment of Fibres. U.S. Patent.

[25] Qing L., Yu K., Mu R., Forth J.P. (2019). Uniaxial Tensile Behavior of Aligned Steel Fibre Reinforced Cementitious Composites. Mater. Struct..

[26] da Silva Brito I., Alan Strauss Rambo D., Martini S., Pícolo Salvador R., Fabrízio de Menezes Freitas M. (2021). Flexural Behavior of HPFRCC: Enhancing Post-Crack Strength and Toughness by Magnetic Alignment of the Reinforcement. Constr. Build. Mater..

[27] Cao G., Qing L., Mu R., Wang L., Yang Z., Zhang J. (2022). Characterization of Toughness Enhancement of Aligned Steel Fibers in Cement-Based Composites Based on DDM Method and *Meso*-Scale Simulation. Constr. Build. Mater..

[28] Michels J., Gams M. (2016). Preliminary Study on the Influence of Fibre Orientation in Fibre Reinforced Mortars. Građevinar.

[29] Wijffels M.J.H., Wolfs R.J.M., Suiker A.S.J., Salet T.A.M. (2017). Magnetic Orientation of Steel Fibres in Self-Compacting Concrete Beams: Effect on Failure Behaviour. Cem. Concr. Compos..

[30] Abrishambaf A., Pimentel M., Nunes S. (2017). Influence of Fibre Orientation on the Tensile Behaviour of Ultra-High Performance Fibre Reinforced Cementitious Composites. Cem. Concr. Res..

[31] Abavisani I., Rezaifar O., Kheyroddin A. (2018). Alternating Magnetic Field Effect on Fine-Aggregate Steel Chip-Reinforced Concrete Properties. J. Mater. Civ. Eng..

[32] Ferrández D., Saiz P., Morón C., Dorado M.G., Morón A. (2019). Inductive Method for the Orientation of Steel Fibers in Recycled Mortars. Constr. Build. Mater..

[33] Hajforoush M., Kheyroddin A., Rezaifar O., Kioumarsi M. (2021). The Effects of Uniform Magnetic Field on the Mechanical and Microstructural Properties of Concrete Incorporating Steel Fibers. Sci. Iran.

[34] Javahershenas F., Gilani M.S., Hajforoush M. (2021). Effect of Magnetic Field Exposure Time on Mechanical and Microstructure Properties of Steel Fiber-Reinforced Concrete (SFRC). J. Build. Eng..

[35] Mu R., Dong R., Liu H., Chen H., Cheng Q., Fan C. (2021). Preparation of Aligned Steel-Fiber-Reinforced Concrete Using a Magnetic Field Created by the Assembly of Magnetic Pieces. Crystals.

[36] Ahmad I., Qing L., Khan S., Cao G., Ijaz N., Mu R. (2021). Experimental Investigations on Fracture Parameters of Random and Aligned Steel Fiber Reinforced Cementitious Composites. Constr. Build. Mater..

[37] Khan S., Qing L., Ahmad I., Mu R., Bi M. (2022). Investigation on Fracture Behavior of Cementitious Composites Reinforced with Aligned Hooked-End Steel Fibers. Materials.

[38] Carrera K., Künzel K., Konrád P., Sovják R., Papež V., Mára M., Fornůsek J., Kheml P. (2022). Concrete Lintels Reinforced with Steel Fibres Oriented by a Magnetic Field. Acta Polytech..

[39] Al Rifai M.M., Sikora K.S., Hadi M.N.S. (2024). Magnetic Alignment of Micro Steel Fibers Embedded in Self-Compacting Concrete. Constr. Build. Mater..

[40] Chen X., Liu Y., Mu R., Chen J., Zhang G., Wang X., Qing L., Liu Y. (2024). Improving Reinforcement Efficiency of Aligned Steel Fibre on Cement-Based Composites by Hybridizing Polypropylene Fibre. J. Build. Eng..

[41] Cheol Lee S., Hwan Oh J., Yeol Cho J. (2015). Fiber Orientation Factor on Rectangular Cross-Section in Concrete Members. Int. J. Eng. Technol..

[42] Varzavand S. (1993). Flexural and Tensile Characteristics of Steel and Polypropylene Fiber Hybrid Reinforced Concrete Composite. Ph.D. Thesis.

[43] Villar V.P., Medina N.F. (2018). Alignment of Hooked-End Fibres in Matrices with Similar Rheological Behaviour to Cementitious Composites through Homogeneous Magnetic Fields. Constr. Build. Mater..

[44] (2002). Concrete-Part 1: Specification, Performance, Production and Conformity.

[45] (2010). Methods of Testing Cement—Part 6: Determination of Fineness.

[46] (2016). Methods of Testing Cement—Part 1: Determination of Strength.

[47] (1999). Methods of Test for Mortar for Masonry—Part 3: Determination of Consistence of Fresh Mortar (by Flow Table).

[48] Nováková K., Carrera K., Konrád P., Künzel K., Papež V., Sovják R. (2022). Simulations of the Behaviour of Steel Ferromagnetic Fibres Commonly Used in Concrete in a Magnetic Field. Materials.

[49] Cheng Z., Zhao H., Long G., Yang K., Chen M., Wu Z. (2023). The Mechanical Characteristics of High-Strength Self-Compacting Concrete with Toughening Materials Based on Digital Image Correlation Technology. Materials.

[50] (2008). Test Method for Metallic Fibre Concrete-Measuring the Flexural Tensile Strength (Limit of Proportionality (LOP), Residual).

[51] Klun M., Bosiljkov V., Bokan-Bosiljkov V. (2021). The Relation between Concrete, Mortar and Paste Scale Early Age Properties. Materials.

[52] Chyliński F. (2024). Microstructural Assessment of Pozzolanic Activity of Ilmenite Mud Waste Compared to Fly Ash in Cement Composites. Materials.

[53] Alberti M.G., Enfedaque A., Gálvez J.C. (2018). A Review on the Assessment and Prediction of the Orientation and Distribution of Fibres for Concrete. Compos. Part B Eng..

[54] Zakrzewski M., Gancarz M., Tvrdá K., Laskowska-Bury J., Domski J. (2023). Comparative Analysis of Waste, Steel, and Polypropylene Microfibers as an Additive for Cement Mortar. Materials.

[55] (2005). Standard Test Method for Flexural Performance of Fiber-Reinforced Concrete (Using Beam with Third-Point Loading).

